# A narrative analysis of common international public health guidelines for drowning prevention with a focus on flood-related drowning

**Published:** 2022-12

**Authors:** Hadis Amiri, Vahid Yazdi-Feyzabadi

**Affiliations:** ^*a*^ Health in Emergencies and Disasters Research Center, Institute for Futures Studies in Health, Kerman University of Medical Sciences, Kerman, Iran.; ^*b*^ Health Services Management Research Center, Institute for Futures Studies in Health, Kerman University of Medical Sciences, Kerman, Iran.

## Abstract

**Background:**

Each day, more than forty people die of drowning every hour. Drowning prevention is usually a concern in many countries. Governments have developed strategies and guidelines to prevent drowning. This study aims to review four different drowning prevention documents.

**Methods:**

A qualitative review was conducted using the document review method in which four documents were identified (1. Global Report on Drowning: Preventing a Leading Killer by WHO, 2. Preventing Drowning: An Implementation Guide by WHO, 3. Drowning Prevention Strategies – International Life Saving Federation, and 4. The UK Drowning Prevention Strategy 2016-2026). They were then analyzed based on the READ framework.

**Results:**

The WHO report recommends the six best ways to reduce drowning. Preventing Drowning: An Implementation Guide introduced six interventions to prevent drowning and four strategies to support drowning prevention interventions. The UK Drowning Prevention Strategy 2016-2026 specified five aims for drowning prevention and four ways to achieve those aims. All four documents agreed that drowning could be prevented through the following actions: 1) targeted prevention strategies, 2) improved community infrastructure (e.g., water supply, bridges, and levees), 3) public awareness-raising, 4) appropriate policies and legislation, 5) and research identifying best practices and new drowning preventive measures. The documents had different strategies, which in many cases are similar—teaching swimming and water safety skills to school-age children. But ”The Global Report on Drowning: Preventing a Leading Killer by WHO“ suggested developing a separate national water safety plan for each country.

**Conclusions:**

According to the study, we can use common strategies in all four documents to update our national strategies– scaling up these approaches will bring further gains. Regional drowning rates in low- and middle-income countries are up to 3.4 times greater than those in high-income countries. Developing context-sensitive strategies based on the world's successful policies is highly suggested.

**Keywords:**

Narrative analysis, International public health guidelines, Drowning prevention, Flood, Drowning

